# The Role of Antioxidant Activity of Chitosan-*Pinus merkusii* Extract Nanoparticle in against Lead Acetate-Induced Toxicity in Rat Pancreas

**DOI:** 10.1155/2019/9874601

**Published:** 2019-11-28

**Authors:** G. Wardani, Ernawati, K. Eraiko, S. A. Sudjarwo

**Affiliations:** ^1^Faculty of Pharmacy, Hang Tuah University, Surabaya 60115, Indonesia; ^2^Faculty of Health, Muhammadiyah University, Gresik, Indonesia; ^3^Department of Conservative Dentistry, Faculty of Dentistry, Airlangga University, Surabaya 60115, Indonesia; ^4^Department of Pharmacology, Faculty of Veterinary Medicine, Airlangga University, Surabaya 60115, Indonesia

## Abstract

Lead is one of the heavy metals with oxidative stress that causes toxicity in human and animals. The aim of this study was to evaluate the antioxidant activity of Chitosan-*Pinus merkusii* extract nanoparticle on lead acetate-induced toxicity in rat pancreas. Chitosan-*Pinus merkusii* nanoparticles were identified by Particle Size Analysis (PSA) and Scanning Electron Microscope (SEM). The male rats used were divided into a control group (treated with distilled water), lead acetate group (injected with lead acetate at 20 mg/kg BW i.p), and the treatment group (treated orally with Chitosan-*Pinus merkusii* nanoparticle at 150 mg; 300 mg; 600 mg/kg BW and injected with lead acetate at 20 mg/kg BW i.p). Blood samples were taken to measure glucose and insulin level. The pancreas tissues were also collected to evaluate the malondialdehyde (MDA), superoxide dismutase (SOD), glutathione peroxidase (GPx), and histological evaluations of cell damage. The PSA showed that the size of Chitosan-*Pinus merkusii* nanoparticle was 530.2 ± 38.27 nm. The SEM images revealed an irregular shape, and the morphology showed a rough surface. Administration of lead acetate resulted in a significant increase in glucose and MDA levels as well as a decrease in the level of insulin, SOD and GPx when compared with the control group, while that of 600 mg/kg BW of Chitosan-*Pinus merkusii* nanoparticle gave a polar result. The lead acetate induced loss of pancreatic cells normal structure and necrosis, while Chitosan-*Pinus merkusii* nanoparticle inhibited it. It could be concluded that Chitosan-*Pinus merkusii* nanoparticle has a potential to be a powerful agent and may be useful as an antioxidant against free radical-induced oxidative stress and pancreatic cell damage mediated by lead acetate intoxication.

## 1. Introduction

Lead is one of the heavy metals that cause acute or chronic health impacts on human and animals. This toxicity may affect various organs such as the heart [1], liver [2], testis [3], kidney [4], and pancreas [5], and other systems in the body [6, 7]. The presence of lead in the pancreas is associated with oxidative stress which has been reported to be one of the possible mechanisms involved in lead pancreatic toxicity [5].

In living systems, the pancreas is considered to be highly sensitive to toxic agents. The association between lead exposure and the risk of non-insulin-dependent diabetes mellitus is a relatively new finding. Several researchers have shown that lead can cause pancreatic *β* cell damage, suppress insulin secretion, increase glucose intolerance, and have diabetogenic effects. Lead induces pancreatic *β* cell death via an oxidative stress damage, causing pancreatic *β*-cell dysfunction and apoptosis or necrosis [5, 7].

This can happen through the oxidative stress which disrupts the balance existing between the capacity of antioxidants to clean and propagation of Reactive Oxygen Species (ROS) in the pancreatic cell [8, 9]. There is a possible increment in the (ROS) such as superoxide ion (O_2_^−^), hydroxyl radical (OH^−^), nitrogen oxide (NO), hydroperoxides (HO_2_), and hydrogen peroxide (H_2_O_2_) generated, as well as a decrease in the antioxidants such as catalase, SOD, and GPx produced [8, 10]. This imbalance may promote the induction of lipid peroxidation, proteins, and DNA damage, leading to the death of pancreatic cells via apoptosis or necrosis [11, 12].

Malondialdehyde (MDA) level is measured to know the level of lipid peroxidation since it is its byproduct. The elevation of MDA concentration is due to the increased peroxidation of lipid membranes and it is an indicator of oxidative stress [13].

It was discovered that the activities of antioxidant or the act of inhibiting free radicals' generation can help in protecting the pancreas from toxicity induced by lead acetate. Therefore, this makes it possible to use antioxidants as protective agents when such situation arises [5, 14]. However, natural products can be used as a good alternative because of their low costs, availability, and lack of undesirable side effects [14, 15].

This study was focused on the protective effect of natural products or herbal medicine with antioxidant properties of Chitosan-*Pinus merkusii* in reducing free radical-induced pancreatic cell damage. It has been demonstrated that *Pinus* plant has phytochemicals such as alkaloids, polyphenols, flavonoids, lignans, triterpenes, sterols, glycosides, triterpenoids, and saponins [16]. Recent research activities have also shown that it is an important source of pycnogenol containing proanthocyanidins (procyanidins) [17, 18]. These are potent as antioxidants, antibacterial, antiallergic, anti-inflammatory, cardioprotective, immune-stimulating, antiviral agents, and also used in estrogenic activities [19–21].

In recent years, the synthesis of natural product nanoparticles has become an interesting topic in nanoscience and nanobiotechnology [22]. Several research studies have shown that they are very important to the biosynthesis of the nanoparticles, especially in large-scale [23]. In medicine, natural products like chitosan are used as drug carriers because they encapsulate a broad range of therapeutic agents that deliver to the target site efficiently. Chitosan is biocompatible and biodegradable properties have led to significant research especially towards biomedical and pharmaceutical applications, such as drug delivery, tissue engineering, wound-healing dressing, and superior regenerative medicine, currently one of the most quickly growing fields in the area of health science. Chitosan nanoparticles are acting as an excellent drug carrier because capable of moving through various biological barriers (like brain barrier) bringing drugs to the target site enhancing its efficacy [24–26]. They have drawn the attention of researchers for their controlled drug release properties and are used in both in-vitro and in-vivo applications [26]. It is nontoxic and also known to possess antibacterial, antioxidant, antihyperlipidemic, antiulcus pepticum, antidiabetic, anti-HIV, anti-inflammatory, drug delivery, and immunoenhancing properties that make it an ideal delivery agent for applications in medicine [27–29]. Therefore, the objective of this study was to investigate the antioxidant activity of the Chitosan-*Pinus merkusii* extract nanoparticle on lead acetate-induced pancreatic cell damage in Wistar albino rats.

## 2. Materials and Methods

### 2.1. Ethical Approval

It is important to point out that the processes of the experiment were adequately checked by the Ethical Clearance Committee for preclinical research, Faculty of Dentistry, Airlangga University and an ethical clearance with the No.200/HRECC.FODM/VII/2018 dated July 31, 2018 was awarded.

### 2.2. Preparation of Chitosan-*Pinus merkusii* Extract Nanoparticles

The Chitosan-*P. merkusii* extract nanoparticle was prepared using ionic gelation with sodium tripolyphosphate (TPP) anions [1, 11]. The 0.2% w/v concentrations of chitosan solutions were prepared in 0.1% v/v glacial acetic acid and filtered. The TPP solution (0.1% w/v) was prepared in deionized water. *P. merkusii* extract 0.4% w/v in 70% ethanol was added to the solution (0.2% w/v) under constant stirring. The mixture was then sonicated for 5 min and the TPP solution was added in drop while it was continuously stirred. A ratio of 2 : 4 : 1 was maintained for chitosan, *P. merkusii*, and TPP solution respectively throughout the experiment. The supernatant obtained was subjected to ultracentrifugation at 25000 rpm for 20 min to sediment the Chitosan-*P. merkusii* conjugated nanoparticles, which were later subjected to further characterization.

### 2.3. Characterization of Nanoparticles by SEM and PSA

The surface morphological features such as particle size, shape, and topography were observed using the SEM. The particle size was determined using PSA (Horiba LA 900, Japan).

### 2.4. Experimental Animal

The male Wistar rats used in this experiment averagely weighed around 200–250 g (2.5–3 months). They were gotten from the Gadjah Mada University, Yogyakarta, Indonesia and placed in plastic cages in a room with controlled temperature of 26 ± 2°C and 12 h alternate light and dark cycles. They were spontaneously given tap water and fed with standard commercial feeds.

### 2.5. Experimental Design

The 50 male rats used as samples were divided into five groups: Control group (where they fed with distilled water daily), lead acetate group (injected with lead acetate solution, i.p., at a dose of 20 mg/kg BW for 7 consecutive days), and the treatment group (given the Chitosan-*P. merkusii* extract nanoparticle at 150 mg, 300 mg, and 600 mg/kg BW orally once in a day for 11 days, and on the 4^th^ day, injected with lead acetate solution, i.p., at a dose of 20 mg/kg BW 1 h after the usual dosage). On the 11^th^ day, blood samples of the rats were taken by cardiac puncture in order to measure glucose and insulin levels. Glucose was measured using a glucometer (Roche Diagnostics Deutschland GmbH, Mannheim, Germany), while Serum insulin levels were determined using an Insulin ELISA Assay Kit [5]. Furthermore, the rats were sacrificed and their pancreas tissues were used for the analysis of MDA and antioxidant enzymes (SOD and GPx). The measurement of the MDA was conducted in the supernatant of homogenized pancreas tissue through the application of the thiobarbituric acid method. The unit used is nanomoles MDA/g tissue [28]. The evaluation of the SOD level was conducted using a detection kit at 505 nm through a standard curve with reference to the instructions from the manufacturer. The unit used is U/mg protein. The measurement of the activity of GPx was conducted spectrophotometrically against blank at 340 nm through the use of a detection kit by following the directives of the manufacturer and the results were expressed as U/mg protein [30].

The pancreas was also fixed in a 10% neutral-buffered formalin solution in order to conduct a histopathological evaluation of the pancreas damage [5].

### 2.6. Statistical Analysis

Data were presented in the form of mean ± standard deviation. *Post hoc* test was conducted through the use of a One-way ANOVA and the groups were statistically compared using a LSD test through the application of SPSS version 17.0 (SPSS Inc, Chicago, USA).

## 3. Results

### 3.1. Characterization of Chitosan-*Pinus merkusii* Extract Nanoparticles by SEM

**Figure 1:**
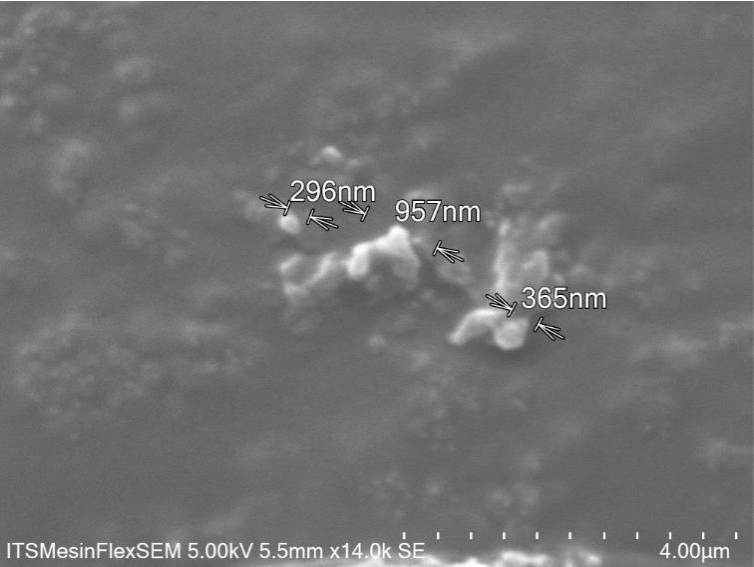
Scanning electron microscope images of Chitosan-*Pinus merkusii* extract nanoparticle.

SEM images gotten from the analysis revealed that it has a rough surface and an irregular shape (Figure 1).

### 3.2. Characterization of Chitosan-*Pinus merkusii* Nanoparticles by PSA

**Figure 2:**
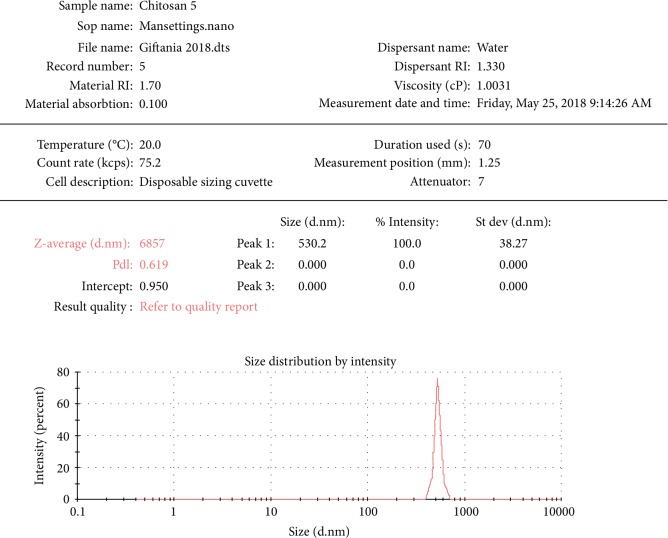
Size distribution of *Chitosan-Pinus merkusii* extract nanoparticles by Particle Size Analysis.

The average particle size gotten from the PSA (Particle size analysis) was 530.2 ± 38.27 nm as shown in Figure 2.

### 3.3. Effects of Chitosan-*Pinus merkusii* Extract Nanoparticles on Lead Acetate-Induced Changes in the Blood Glucose and Serum Insulin

This analysis was conducted for the purpose of evaluating the effect of the extracts on the rats treated with the lead acetate. It was found that the glucose level in the blood increased, while the serum insulin levels decreased significantly (*P* < 0.05) when a comparison is drawn with the control group. It is important to note that this indicates damages in the pancreas. However, the groups pretreated with 600 mg/kg BW of extract nanoparticles showed the exact opposite when compared with the lead acetate and control groups (Table 1).

### 3.4. Effects of Chitosan-*Pinus merkusii* Extract Nanoparticles on Lead Acetate-Induced Changes on MDA, SOD, and GPx in Rat Pancreas

Pancreas are damaged through the formation of reactive oxygen species intercellularly which can be enhanced by lead acetate. Therefore, the analysis of the MDA and antioxidants like SOD and GPx levels was conducted. It was found that the group treated with the lead acetate significantly (*P* < 0.05) increased MDA levels, while SOD and GPx were discovered to have decreased when a comparison is drawn with the control group. However, the 600 mg/kg BW extracts pretreated group showed the exact opposite when compare with the lead acetate and control groups as shown in Table 2.

### 3.5. Effects of Chitosan-*Pinus merkusii* Extract Nanoparticles on Lead Acetate Induce Pancreas Damage

**Figure 3:**
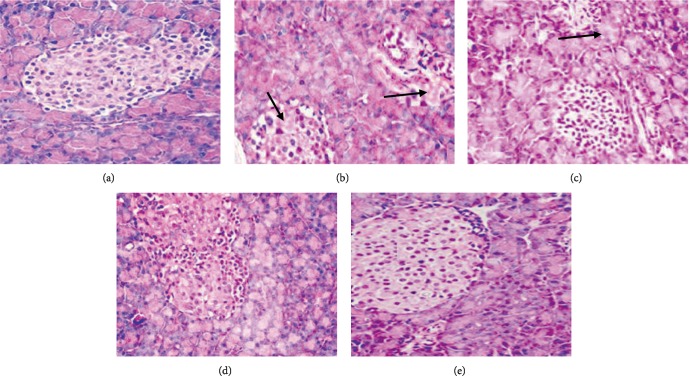
Normal of pancreas sections in control group (a). Histopathological view of pancreas sections in lead acetate treated group shown degenerative and necrotic changes (black arrow) in the islets of Langerhans as compared to control group (b). Rat treated with Chitosan-*Pinus merkusii* extract nanoparticles 600 mg/kg showed regeneration (e), while Chitosan-*Pinus merkusii* extract nanoparticles 300 mg/kg (c), and 150 mg/kg (d) showed degeneration in the islets of Langerhans, using haematoxyline and eosin stain technique (×400).

Light microscopy was used in investigating the histopathological study. The microscopic assessment of the normal pancreas revealed that normal acini and cellular population are present in the islets of Langerhans in the control group while degenerative and necrotic variations as well as the shrinking of islets in the pancreas tissues were observed in the lead acetate-treated group. The pretreatment conducted with 600 mg/kg BW Chitosan-*Pinus * extract nanoparticles significantly prevented histopathological changes from normal (Figure 3).

## 4. Discussion

*Pinus merkusii* extract was encapsulated into chitosan nanoparticle through the use of sodium tripolyphosphate as a cross-linking agent on the ionotropic gelation method, which has more advantages than using extract only [1, 5]. The modification to nanoparticle can improve biodistribution, increase specificity and sensitivity, and reduce pharmacological toxicity [25, 26]. The nanoparticles obtained in this study had a small particle size (530.2 ± 38.27 nm), which may increase its antioxidant activity. The SEM images showed that it has an irregular shape while the morphological surface was observed to be rough.

Reports from different studies conducted revealed that toxicity associated with leads mostly target the pancreas [7, 8]. The result of the experiment revealed that the blood glucose level significantly increased, while that of serum insulin decreased after lead acetate was injected in the samples. This can be associated with the oxidative stress that helps to induce pancreatic islet *β*-cell injuries, overproduction of mitochondrial ROS and lack of enough antioxidant enzymes in the *β*-cells. This is possible because lead acetate produces oxidative that damages the pancreas by enhancing lipid peroxidation and increasing free radical damage [9, 10]. The administration of Chitosan-*Pinus merkusii* nanoparticle 600 mg/kg BW was observed to have improved the blood glucose and insulin levels. This may be associated with its direct action on free radicals of lead acetate to prevent pancreatic cellular damage by maintaining its membrane integrity.

In this research, administering lead acetate increased MDA and induced oxidative damage in the pancreas. It has been discovered that MDA can be used as an indicator of cell membrane injury. Therefore, increasing its levels can enhance lipid peroxidation which can lead to tissue damage and failure of antioxidant defense mechanisms that used in preventing the formation of excessive free radicals [2, 3]. The rats treated with 600 mg/kg BW Chitosan-*Pinus merkusii *extract nanoparticle were found to have prevented the levels to rise when the lead acetate is still in their pancreas. This might be due to the ability of dosage to reduce the accumulation of free radicals because it has been observed to be functioning as a powerful antioxidant and free radical scavenger that can decrease the MDA level perturbed by lead acetates. The findings suggest that Chitosan-*Pinus merkusii* extract nanoparticle could attenuate oxidative stress by decreasing the lipid peroxidation (MDA level) in the lead-treated pancreas.

The presence of oxidative stress in the toxicity of heavy metals could be measured through the levels of antioxidant enzyme levels because they both form a reciprocally supporting defense mechanism against ROS. Different scholars and researchers have revealed that lead is highly reactive with the SH groups of different enzymes and by reacting with them, it affects their antioxidant tendencies [7, 31]. The results of the experiment revealed that there was a decrease in the activities of SOD and GPx in the samples when they were treated with lead acetate. This happened because lead was able to increase the ROS and deplete the antioxidants reserves thereby causing free radical damage to the tissues. However, the administration of the extract increased the activities of the enzymes. This is possible because of the ability of the extract to suppress the free radicals accumulated, which helps in protecting the pancreas. Moreover, the extracts-altered antioxidant defense system was discovered to be the cause for the lipid peroxidation reduction.

It has been reported by some researchers that Histopathological results can show structural changes in pancreas tissue of heavy metal toxicity such as lead acetate. The analysis conducted in this study revealed renal cell damage (necrosis). It was considered mild in the groups treated with the extract. This can be associated with the report that lead toxicity can cause excessive production of ROS, thereby causing an imbalance between the production of oxidants and the defense systems of antioxidant. This may promote the induction of lipid peroxidation, proteins, and DNA damage and also lead to the death of the pancreatic cell via apoptosis or necrosis [7, 12]. However, the administration of Chitosan-*Pinus merkusii *extract nanoparticle lessened the effects of lead acetate-induced pancreastoxicity possibly through its antioxidant mechanisms.

## 5. Conclusion

It could be concluded that Chitosan-*Pinus merkusii* extract nanoparticle may exert its protective actions against lead acetate-induced pancreas toxicity in rats, through its antioxidant mechanisms. Chitosan-*Pinus merkusii* extract nanoparticle can be a future natural product for counteracting the lead acetate intoxication. These results showed that Chitosan-*Pinus merkusii* extract nanoparticle has a potential pancreoprotective effect in a dose-dependent manner that minimizes or diminishes the pancreas toxicity effect of lead acetate.

## Figures and Tables

**Table 1:** Effect of Chitosan-*Pinus merkusii* extract nanoparticle on lead acetate-induced changes on the blood glucose and serum insulin.

Groups	Mean ± SD	
	Blood glucose (mg/dl)	Serum insulin (*μ*U/ml)
Control	98.21^a^ ± 6.23	68.73^a^ ± 4.75
Lead acetate groups	276.17^b^ ± 9.43	41.44^b^ ± 5.83
Chitosan-*P. merkusii* 150 mg/kg BW	288.31^b^ ± 7.67	39.62^b^ ± 4.91
Chitosan-*P. merkusii* 300 mg/kg BW	263.62^b^ ± 8.92	48.20^b^ ± 4.64
Chitosan-*P. merkusii* 600 mg/kg BW	168.57^c^ ± 10.23	52.39^c^ ± 3.85

^a,b,c^Different superscript within each column indicate significant difference between the means (*P* < 0.05).

**Table 2:** Effects of Chitosan-*Pinus merkusii* extract nanoparticle on lead acetate-induced changes in the superoxide dismutase, glutathione peroxidase and malondialdehyde.

Groups	Means ± standard deviation		
	SOD (U/mg)	GPx (U/mg)	MDA (nmol/mg)
Control	26.73^a^ ± 2.01	53.37^a^ ± 6.16	7.29^a^ ± 0.87
Lead acetate groups	16.97^b^ ± 1.87	34.55^b^ ± 4.62	12.35^b^ ± 1.15
Chitosan-*P. merkusii* 150 mg/kg BW	15.88^b^ ± 2.52	33.91^b^ ± 6.47	11.42^b^ ± 0.93
Chitosan-*P. merkusii* 300 mg/kg BW	17.47^b^ ± 1.61	35.81^b^ ± 3.29	10.87^b^ ± 0.91
Chitosan-*P. merkusii* 600 mg/kg BW	21.08^c^ ± 1.03	44.27^c^ ± 4.72	9.11^c^ ± 0.76

^a,b,c^Different superscript within each column indicate significant difference between the means (*P* < 0.05).

## Data Availability

The data used to support the findings of this study are included within the article.
